# Micro‐ and macrovascular complications and risk factors for foot ulceration and amputation in individuals receiving dialysis with and without diabetes

**DOI:** 10.1002/edm2.305

**Published:** 2021-10-17

**Authors:** Dea Haagensen Kofod, Thomas Peter Almdal, Vibeke Rømming Sørensen, Bo Feldt‐Rasmussen, Mads Hornum

**Affiliations:** ^1^ Department of Nephrology Rigshospitalet University of Copenhagen Copenhagen Denmark; ^2^ Department of Endocrinology Rigshospitalet University of Copenhagen Copenhagen Denmark; ^3^ Department of Clinical Medicine Faculty of Health and Medical Sciences University of Copenhagen Copenhagen Denmark

**Keywords:** diabetes complications, diabetes mellitus, diabetic foot, dialysis, end‐stage renal disease, epidemiology

## Abstract

**Introduction:**

This study examined the prevalence of microvascular and macrovascular complications in people receiving dialysis with and without diabetes and investigated independent risk factors for foot ulcers and lower‐extremity amputations.

**Methods:**

We performed a cross‐sectional study of 119 individuals with diabetes and 219 individuals without diabetes receiving chronic dialysis during June 2019 at the Department of Nephrology, Rigshospitalet, University of Copenhagen, Denmark. Effects of diabetes and other risk factors were assessed by log‐binomial regression. Prevalence data were compared with a historical control group of 38 individuals with diabetes receiving dialysis examined in 2004 in the same department.

**Results:**

We found that persons with diabetes had a twofold higher risk ratio of current (unadjusted risk ratio 2.2 [95% CI 1.1, 4.7]) and previous foot ulcer (2.5 [1.7, 3.7]) and a fourfold higher risk ratio of lower‐extremity amputation (4.2 [2.1, 8.6]) in comparison with persons without diabetes (all *p* < .05). Furthermore, persons with diabetes had a 70% increased risk ratio of myocardial infarction (1.7 [1.0–2.8], *p* = .041). In multivariable‐adjusted analysis, current foot ulcer was independently associated with previous foot ulcer (adjusted risk ratio 4.0 [95% CI 1.8, 8.9]), while lower‐extremity amputation was independently associated with diabetes (3.8 [1.8, 8.2]) and male sex (4.1 [1.5, 11.3]) (all *p* < .01).

**Conclusions:**

Individuals with diabetes receiving dialysis had a higher prevalence of foot ulcer, lower‐extremity amputation and myocardial infarction compared to individuals without diabetes. Previous foot ulcer was the most important risk factor for current foot ulcer, while diabetes and male sex were important risk factors for lower‐extremity amputation.

## Graphical abstract

Over the past decades, there has been a general improvement in the prognosis of people with diabetes; however, it is unclear what the consequences of these improvements are in relation to complications among people receiving dialysis. This study examined the prevalence of micro‐ and macrovascular complications in people receiving dialysis with and without diabetes, and found that individuals with diabetes had a higher prevalence of foot ulcer, amputation and myocardial infarction compared to individuals without diabetes.

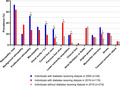

## 1 INTRODUCTION

Diabetic nephropathy remains the leading cause of end‐stage renal disease (ESRD) in most countries.^1^ The combination of diabetes and ESRD is associated with a high prevalence of macrovascular complications, including a particularly high risk of adverse cardiovascular outcomes.^2^, ^3^ Microvascular complications other than kidney failure, that is retinopathy and neuropathy, are also very frequent in this population.^3^ In a study from 2007, we reported a high prevalence of current foot ulcers, lower‐extremity amputations and advanced diabetic eye complications in individuals with diabetes receiving dialysis, as compared to both individuals receiving dialysis without diabetes and individuals with diabetes with normal kidney function.^4^

Since this study was performed, there has been a general improvement in the prognosis of people with diabetes in terms of reduced complication and mortality rate.^5^, ^6^, ^7^ These improvements also apply to individuals with diabetic nephropathy without ESRD, and an improved survival and renal prognosis for this population suggest that the individuals who reach ESRD have had diabetes for a longer duration than previously.^8^, ^9^ It is unclear what the consequences of these changes are in relation to the prevalence of micro‐ and macrovascular complications among people with ESRD receiving dialysis.

In the present study, we assessed the prevalence of micro‐ and macrovascular complications in a cross‐sectional study of individuals receiving dialysis with and without diabetes, and examined whether the prevalence of complications has changed since the above‐mentioned study. Furthermore, we examined independent risk factors for current foot ulcers and lower‐extremity amputations.

## 2 METHODS

### 2.1 Study population

We conducted a cross‐sectional study of individuals receiving chronic dialysis therapy (haemodialysis and peritoneal dialysis) during June 2019 at the Department of Nephrology, Rigshospitalet, University of Copenhagen, Copenhagen, Denmark. Individuals were identified using the department's records of people receiving chronic dialysis therapy. Inclusion criteria were chronic dialysis therapy for more than 3 months and age ≥18 years. People living in Greenland were excluded as we did not have access to their medical records. Subsequently, individuals were classified according to diabetes status into the following two groups: (1) the group with diabetes that included individuals with a previous or current diagnosis of diabetes, and (2) the group without diabetes with no known diagnosis of diabetes.

### 2.2 Data collection and definitions

Data were collected using the unique personal identification number assigned to all persons in Denmark. Data regarding the duration of ESRD were obtained from the Danish Society of Nephrology National Registry. Data on diabetes diagnosis and all other study variables were collected by reviewing the individuals’ electronic medical records.

Data regarding clinical characteristics included sex, age, body mass index (BMI), current smoking status, total cholesterol, hypertension (as defined by the treatment of ≥1 anti‐hypertensive medication), systolic and diastolic blood pressure, anti‐hypertensive treatment, duration of ESRD, dialysis modality, haemodialysis access and dialysis adequacy (Kt/V). Furthermore, for the group with diabetes, data included diabetes type, duration of diabetes, HbA_1c_ level, glucose‐lowering treatment, hospitalization with hypoglycaemia within the past year, and whether individuals currently attended a podiatrist.

The outcome variables were microvascular complications defined as current foot ulcer, previous foot ulcer, previous lower‐extremity amputation, current or previous Charcot foot, background retinopathy, proliferative retinopathy, maculopathy, visual acuity below 0.3 and macrovascular complications defined as a history of atrial fibrillation or atrial flutter, angina pectoris, myocardial infarction, stroke and transient ischaemic attack. Additionally, the temporal relationship between first foot ulcer or first amputation and the start of dialysis was recorded. Outcome data were collected on both individuals with and without diabetes, except Charcot foot and eye complications, as it only concerns individuals with diabetes.

Lower‐extremity amputation was defined as a minor amputation if it was distal to the ankle and a major amputation if through or proximal to the ankle. For individuals who had undergone multiple amputations, the highest level of amputation was used in the analysis. Prevalence of background or proliferative retinopathy, maculopathy and visual acuity below 0.3 was based on the individual's poorest eye.

The current prevalence of micro‐ and macrovascular complications was compared with a historical control group of 38 individuals with diabetes receiving dialysis examined in 2004 in the same department.^4^

### 2.3 Data analysis

All analyses were performed in SPSS version 25 (SPSS Inc., Chicago, IL, USA). *p* Value <.05 was considered statistically significant. The mean value (standard deviation) was applied for normally distributed data and median (interquartile range) for non‐normally distributed data.

BMI was calculated as weight in kilograms divided by height squared in metres. For individuals with major amputations, BMI was adjusted to account for the estimated weight of the missing limb. In this case, we used the estimated body weight calculated as weight divided by 1 minus *P*, where *P* designates the estimated percentage of total body mass set to *p* = .0326 (3.26%) for below‐knee amputations and *p* = .0996 (9.96%) for above‐knee amputations.^10^

The two‐sample *t* test was used to compare continuous normally distributed data and Mann‐Whitney *U* test to compare continuous non‐normally distributed data between study groups. Pearson's chi‐Square and Fisher's exact tests were used to compare categorical data, including differences in the prevalence of micro‐ and macrovascular complications between individuals with diabetes in 2019 and individuals with diabetes in 2004.

Effects of diabetes and other risk factors were assessed by log‐binomial regression models. Potential risk factors were assessed individually in univariable log‐binomial regression models. Risk factors associated with the dependent variable in univariable analysis (defined as P value <0.3) were then included in the multivariable‐adjusted log‐binomial regression analysis. Risk estimates were presented as a risk ratio (RR) with 95% confidential interval (CI) and P value.

Individuals with bilateral major amputations (five individuals in the 2019 group with diabetes) were excluded from analyses regarding podiatrist attendance and current foot ulcer. Individuals with missing eye examination (16 individuals in the 2019 group with diabetes) were excluded from analyses regarding retinopathy, maculopathy and visual acuity.

The study was performed as an audit, which required no formal ethics permission, but was approved by the hospital management of Rigshospitalet (approval 2019‐05‐27).

## 3 RESULTS

**FIGURE 1:**
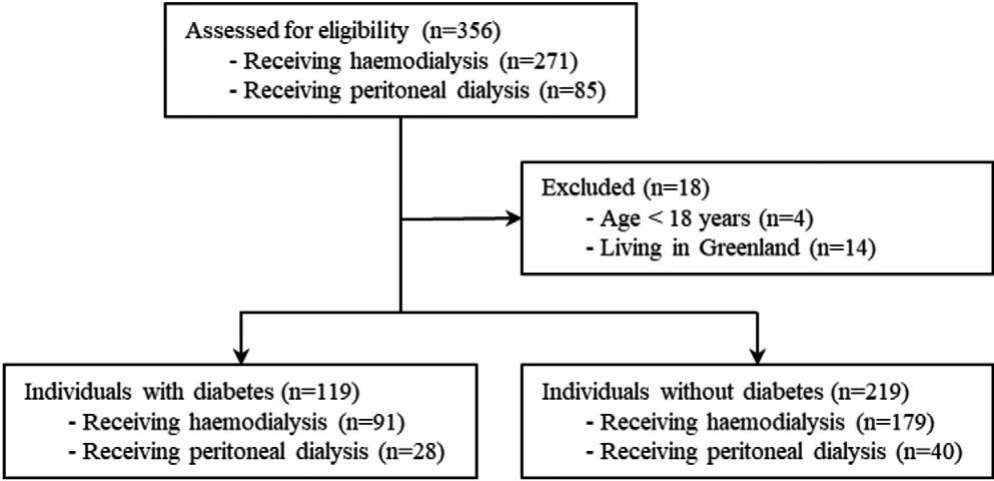
Flow diagram of study groups

Figure 1 shows the flow diagram of the study groups. Of the 356 persons assessed for eligibility, 338 eligible individuals were included in the analysis data set, 119 (35%) with diabetes and 219 (65%) without diabetes.

Clinical characteristics and data on glucose‐lowering and anti‐hypertensive treatment of people in each category are reported in Tables 1 and 2, respectively. Regarding individuals examined in 2019, those with diabetes tended to be older, have a higher BMI, lower total cholesterol and were less likely to smoke compared to those without diabetes. Compared to individuals with diabetes in 2004, those with diabetes in 2019 were more likely to have type 2 diabetes, higher BMI, lower HbA_1c_ level and were less likely to smoke.

**TABLE 1:** Clinical characteristics of individuals with and without diabetes receiving dialysis in 2019 and individuals with diabetes receiving dialysis in 2004.

	2019			2004	
	Diabetes (*n* = 119)	No diabetes (*n* = 219)	*p* Value^a^	Diabetes (*n* = 38)	*p* Value^b^
Male sex	81 (68)	146 (67)	.793	26 (68)	.967
Age, years	70 (58–76)	61 (46–73)	.**000**	64 ± 13	.143
Body mass index, kg/m^2^	27.2 (24.0–30.8)	23.8 (21.8–26.4)	.**000**	25.2 ± 4.0	.**009**
Currently smoking	19 (16)	63 (29)	.**009**	12 (32)	.**035**
Total cholesterol, mmol/L	3.8 (3.3–5.1)	4.6 (3.8–5.6)	.**000**	4.5 ± 1.2	.088
Hypertension	86 (72)	148 (68)	.372	28 (74)	.865
Systolic blood pressure, mmHg	142 ± 21	138 ± 21	.116	143 ± 20	.783
Diastolic blood pressure, mmHg	76 ± 12	79 ± 14	.**046**	74 ± 10	.105
Diabetes					
Type 1 diabetes	13 (11)	‐		11 (29)	.**007**
Duration of diabetes, years	19 (12–26)	‐		15 (7–22)	.074
Current glucose‐lowering treatment	77 (65)	‐		28 (74)	.306
Hypoglycaemia within the past year	5 (4.2)	‐		‐	
HbA_1c_, mmol/mol	46 (37–58)	‐		50 (41–66)	.**049**
HbA_1c_, %	6.3 (5.6–7.5)	‐		6.8 (5.9–8.1)	.050
Attend podiatrist	76 (67)	‐		31 (82)	.081
Dialysis					
Duration of ESRD, years	1.9 (1.0–3.8)	2.2 (0.8, 5.1)	.196	1.9 (1.2–3.9)	.516
Dialysis modality (HD)	91 (77)	179 (82)	.249	32 (84)	.313
HD access (fistula)	57 (63)	118 (54)	.593	16 (50)	.211
Kt/V/dialysis (HD)	1.2 (1.0–1.4)	1.2 (1.0–1.3)	.505	1.6 (1.4–1.7)	.**000**
Kt/V/week (PD)	2.6 (1.9–2.9)	2.5 (2.0–2.9)	.988	2.3 (2.1–2.8)	.829

Note

Data are presented as mean ± SD, median (interquartile range) or *n* (%). ESRD denotes end‐stage renal disease, HD haemodialysis, and PD peritoneal dialysis. Five individuals with diabetes in 2019 had bilateral major amputations and were not included in the data regarding attending podiatrist. Total cholesterol data were missing for 49 individuals without diabetes in 2019.

The use of bold values indicates a *p*‐value below .05.

^a^ For difference between individuals with diabetes in 2019 and individuals without diabetes in 2019.

^b^ For difference between individuals with diabetes in 2019 and individuals with diabetes in 2004.

**TABLE 2:** Glucose‐lowering and anti‐hypertensive treatment

	2019			2004	
	Diabetes (*n* = 119)	No diabetes (*n* = 219)	*p* Value^a^	Diabetes (*n* = 38)	*p* Value^b^
Insulin therapy	71 (60)	‐		21 (55)	.632
Rapid acting	1 (0.8)				
Long acting	29 (24)				
Rapid and long acting	28 (24)				
Premixed	11 (9.2)				
Pump	2 (1.7)				
Oral glucose‐lowering medication	13 (11)	‐		8 (21)	.110
Dipeptidyl peptidase−4 inhibitors	12 (10)				
Sulfonylurea	2 (1.7)				
Anti‐hypertensive treatment	86 (72)	148 (68)	.372	28 (74)	.865
Number of anti‐hypertensive drugs	2 (0–3)	2 (0–3)	.339	1 (0–2)	.306
1‐drug	18 (15)	27 (12)		12 (32)	
2‐drug	30 (25)	53 (24)		8 (21)	
3‐drug	30 (25)	40 (18)		6 (16)	
4‐drug	6 (5.0)	18 (8.2)		2 (5.3)	
5‐drug	2 (1.7)	8 (3.7)		0	
6‐drug	0	2 (0.9)		0	
Anti‐hypertensive drug class					
Loop diuretics	51 (43)	87 (40)			
Thiazides	1 (0.8)	2 (0.9)			
Potassium‐conserving diuretics	0	4 (1.8)			
Calcium channel blockers	53 (45)	88 (40)			
ACE inhibitors	7 (5.9)	13 (5.9)			
Angiotensin II receptor blockers	20 (17)	32 (15)			
Beta blockers	40 (34)	81 (37)			
Alpha blockers	5 (4.2)	12 (5.5)			
Combined alpha beta blockers	21 (18)	33 (15)			
Central acting	3 (2.5)	14 (6.4)			
Minoxidil	1 (0.8)	11 (5.0)			

Note

Data are presented as *n* (%) or median (interquartile range).

Abbreviation: ACE, angiotensin‐converting enzyme.

^a^ For difference between individuals with diabetes in 2019 and individuals without diabetes in 2019.

^b^ For difference between individuals with diabetes in 2019 and individuals with diabetes in 2004.

**FIGURE 2:**
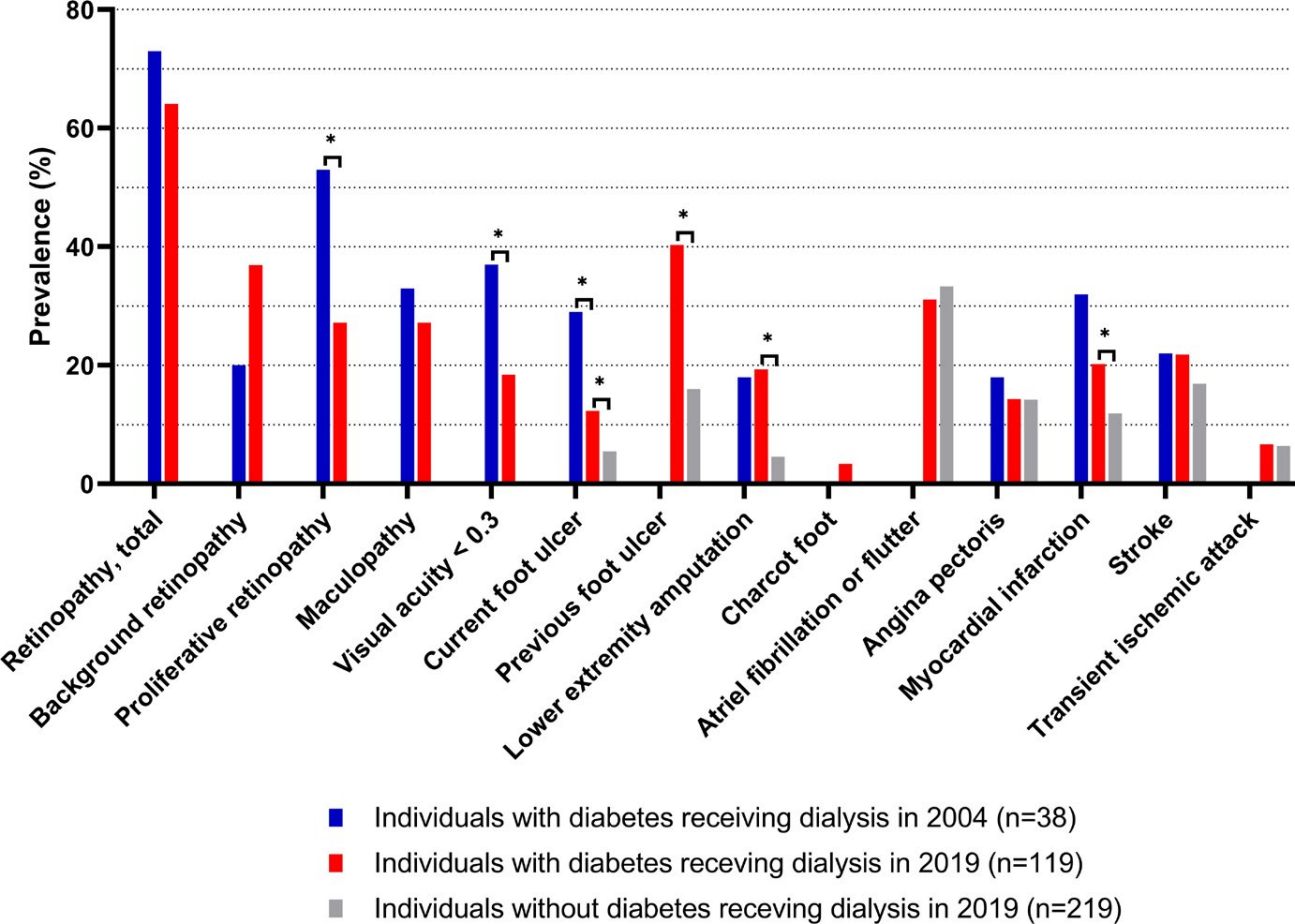
Prevalence of complications in individuals with and without diabetes receiving dialysis in 2019 and individuals with diabetes receiving dialysis in 2004. *p < .05

Figure 2 depicts the current prevalence of micro‐ and macrovascular complications in people with and without diabetes and the prevalence in our historical control group. Comparing individuals with diabetes in 2019 with those in 2004, we found a significant reduction from 29% to 12% in the prevalence of current foot ulcer (*p* = .013), but no reduction in the prevalence of lower‐extremity amputations (*p* = .825). The retinopathy severity level was improved with a significant reduction in the prevalence of proliferative retinopathy from 53% to 27% (*p* = .007), and significantly fewer individuals had a visual acuity below 0.3 with a prevalence reduction from 37% to 18% (*p* = .028). There was no significant difference in the prevalence of total retinopathy (background plus proliferative) (*p* = .387), background retinopathy alone (*p* = .059) or maculopathy (*p* = .476). Furthermore, there was no significant difference in the prevalence of angina pectoris (*p* = .537), myocardial infarction (*p* = .122) or stroke (*p* = .962). In addition, we found no significant difference when performing separate analysis for individuals with type 1 and type 2 diabetes, respectively, with regard to the prevalence of angina pectoris and stroke when comparing individuals with diabetes in 2019 with those in 2004 (data not shown).

The unadjusted risk ratio for micro‐ and macrovascular complications comparing people receiving dialysis in 2019 with and without diabetes is reported in Table 3. People with diabetes had a twofold higher risk ratio of current and previous foot ulcer and a fourfold higher risk ratio of lower‐extremity amputation in comparison with people without diabetes. Furthermore, the risk ratio of myocardial infarction was 70% increased among people with diabetes compared to those without.

**TABLE 3:** Unadjusted risk ratio (RR) of complications comparing individuals with and without diabetes receiving dialysis in 2019

	RR (95% CI)	*p* Value
Current foot ulcer	2.24 (1.07, 4.68)	.**032**
Previous foot ulcer	2.52 (1.74, 3.67)	.**000**
Lower‐extremity amputation	4.23 (2.09, 8.59)	.**000**
History of		
Angina pectoris	1.01 (0.58, 1.75)	.974
Myocardial infarction	1.70 (1.02, 2.82)	.**041**
Stroke	1.29 (0.83, 2.03)	.262
Transient ischaemic attack	1.05 (0.45, 2.44)	.906
Atrial fibrillation or flutter	0.93 (0.67, 1.29)	.676

Note

The reference group for calculation was the group without diabetes.

The use of bold values indicates a *p*‐value below .05.

Investigated risk factors for a current foot ulcer and lower‐extremity amputation assessed in univariable and multivariable‐adjusted analyses are presented in Tables 4 and 5. Presence of diabetes, previous foot ulcer, increasing age and longer duration of diabetes were statistically significantly associated with an increased risk ratio of current foot ulcer, while the presence of hypertension was associated with a decreased risk ratio of current foot ulcer in the univariable analyses. In the multivariable‐adjusted analysis, previous foot ulcer, age and hypertension remained significantly related to a current foot ulcer, with previous foot ulcer increasing the risk ratio fourfold. Diabetes lost its significance in the multivariable analysis. For lower‐extremity amputation, univariable analyses showed that the presence of diabetes and male sex were significantly associated with an increased risk ratio, and in multivariable‐adjusted analysis, both were associated with a fourfold increased risk ratio of amputation. Presence of hypertension was, as for current foot ulcers, associated with a significantly decreased risk ratio of amputation, in both univariable and multivariable analyses.

**TABLE 4:** Univariable and multivariable‐adjusted risk ratio (RR) for risk factors associated with current foot ulcer in individuals with and without diabetes receiving dialysis in 2019

	Univariable analysis		Multivariable‐adjusted analysis	
	RR (95% CI)	*p* Value	RR_adj_ (95% CI)	*p* Value
Diabetes	2.24 (1.07, 4.68)	.**032**	1.30 (0.63, 2.71)	.470
Male sex	1.64 (0.68, 3.98)	.270	1.64 (0.71, 3.80)	.245
Age (years)	1.04 (1.01, 1.07)	.**006**	1.03 (1.00, 1.06)	.**032**
Body mass index	0.98 (0.91, 1.06)	.672		
Hypertension	0.28 (0.13, 0.59)	.**001**	0.40 (0.19, 0.85)	.**017**
Duration of ESRD (years)	0.98 (0.88, 1.10)	.721		
Previous foot ulcer	6.18 (2.87, 13.30)	.**000**	3.98 (1.78, 8.90)	.**001**
Duration of diabetes (years)	1.03 (1.00, 1.06)	.**029**		

Note

ESRD denotes end‐stage renal disease. Duration of diabetes excluded individuals without diabetes in the univariable analysis. All variables with *p* value <0.3 in the univariable analysis were included in the multivariable analysis (except duration of diabetes).

The use of bold values indicates a *p*‐value below .05.

**TABLE 5:** Univariable and multivariable‐adjusted risk ratio (RR) for risk factors associated with lower‐extremity amputation in individuals with and without diabetes receiving dialysis in 2019

	Univariable analysis		Multivariable‐adjusted analysis	
	RR (95% CI)	*p* Value	RR_adj_ (95% CI)	*p* Value
Diabetes	4.23 (2.09, 8.59)	.**000**	3.84 (1.80, 8.19)	.**000**
Male sex	3.55 (1.28, 9.84)	.**015**	4.09 (1.48, 11.31)	.**007**
Age (years)	1.02 (0.99, 1.05)	.059	1.00 (0.98, 1.03)	.765
Body mass index	1.04 (0.99, 1.10)	.109	1.03 (0.97, 1.09)	.325
Hypertension	0.37 (0.19, 0.71)	.**003**	0.33 (0.18, 0.60)	.**000**

Note

All variables with *p* value <0.3 in the univariable analysis were included in the multivariable analysis.

The use of bold values indicates a *p*‐value below .05.

**FIGURE 3:**
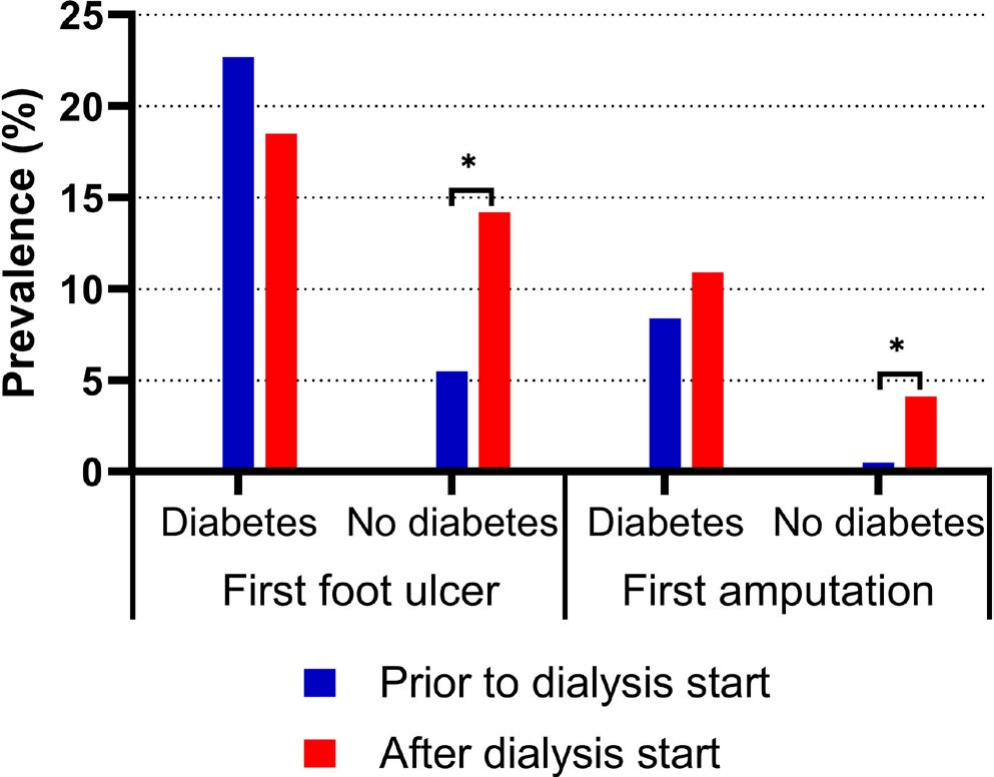
Temporal relationship between first foot ulcer or first lower‐extremity amputation and start of dialysis in individuals with and without diabetes receiving dialysis in 2019. *p < .05

Figure 3 shows the temporal relationship between first foot ulcer or first amputation and the start of dialysis. For the group with diabetes, there was no significant increase or decrease in the incidence of first foot ulcer (*p* = .312) or first amputation (*p* = .376) after the start of dialysis. For the group without diabetes, there was a significantly higher incidence of first foot ulcer (*p* = .000) and first amputation (*p* = .000) after the start of dialysis compared to the incidence prior to dialysis start. Regarding recurrence of foot ulcers, 13 of 14 (93%) individuals with a current foot ulcer in the group with diabetes had a previous foot ulcer, while only four of 12 (33%) without diabetes with current foot ulcer had a previous foot ulcer. Furthermore, individuals with diabetes had a significantly higher prevalence of major amputations compared to individuals without diabetes (9.2 vs 1.4%, *p* = .001).

## 4 DISCUSSION

The present study examined the prevalence of micro‐ and macrovascular complications in people receiving dialysis with and without diabetes. Overall, we found a high prevalence of both micro‐ and macrovascular complications in people receiving dialysis. Comparing complication prevalence between individuals with and without diabetes, individuals with diabetes had a significantly higher risk ratio of current and previous foot ulcer, lower‐extremity amputation and myocardial infarction.

In the multivariable‐adjusted analysis, previous foot ulcer was the single most important risk factor for having a current foot ulcer, increasing the risk ratio fourfold. This is consistent with previous studies reporting a strong correlation between previous foot ulcer and current foot ulcer.^11^, ^12^, ^13^, ^14^, ^15^ Diabetes did not remain an independent risk factor in the multivariable‐adjusted analysis, suggesting that foot ulcers in people receiving dialysis are likely to be related to various risk factors of renal failure, including vascular calcification, peripheral artery disease, anaemia, tissue oedema, infection and immobility. Among previous studies of people receiving dialysis, some report a positive correlation between diabetes and current foot ulcers^12^, ^15^; however, the findings are inconsistent.^13^, ^14^

In contrast to current foot ulcers, both diabetes and male sex were considerable risk factors for lower‐extremity amputation in the multivariable‐adjusted analysis. This discrepancy could partly be explained by the fact that a part was amputated prior to dialysis start, where the impact of diabetes and sex might be of greater importance. Previous studies of lower‐extremity amputations in people receiving dialysis similarly found diabetes and male sex to be independent risk factors of amputations.^12^, ^15^, ^16^ In addition, previous studies found a strong association between previous foot ulcer and amputation,^12^, ^13^, ^15^, ^17^ albeit our data did not allow for such analysis as we did not record the temporal relationship between previous foot ulcers and amputations.

Interestingly, hypertension was a protective factor for both current foot ulcer and amputation. A possible explanation for this could be that our data are biased by the inclusion of individuals with hypotension in our non‐hypertensive population, considering that chronic hypotension has been found to affect 5%–10% of people receiving haemodialysis, and is associated with a poor prognosis.^18^ Chronic hypotension might be caused by diabetes‐ and uraemia‐related cardiovascular autonomic neuropathy, a serious condition that is also associated with increased risk of other complications.^19^, ^20^, ^21^

We found a significant reduction in the prevalence of current foot ulcers and eye complications from 2004 to 2019 among individuals with diabetes receiving dialysis, whereas there was no statistically significant reduction in the prevalence of amputations or cardiovascular and cerebrovascular complications. To our knowledge, this study is the first to document a decreased prevalence of microvascular complications over the past 15 years in people with diabetes receiving dialysis. These results are consistent with observations regarding people with diabetes without ESRD in Denmark, reporting a declining prevalence of foot ulcers and proliferative retinopathy.^8^, ^22^ Several different factors might contribute to a decrease in microvascular complications. These include improved treatment of metabolic risk factors and lifestyle factors, such as reduced smoking. The Steno‐2 trial demonstrated a reduced progression of microvascular complications in people with type 2 diabetes and microalbuminuria as a result of the introduction of multifactorial intervention.^23^ Regarding foot ulcers, increased emphasis on early treatment and continuous follow‐up appears to be of particular importance. To this end, our department started routine check‐ups for foot ulcers in the dialysis unit in the population with diabetes after the study revealed a high prevalence of foot ulcers in 2004. Furthermore, in Denmark, people with diabetes and previous foot ulcer are eligible for a monthly visit to a podiatrist. Consequently, it is likely that ulcers are treated at an earlier stage, which might reduce healing time.^24^

The substantially decreased prevalence of current foot ulcers was not accompanied by a decrease in lower‐extremity amputations. Other studies of people with diabetes with and without ESRD suggest a trend towards a decline in major amputations, while minor amputations remain stable or increase.^25^, ^26^, ^27^ This might be explained by a change in clinical practice that favours earlier minor amputations to avoid major amputations in the future.^25^, ^26^ Data on the distribution of major and minor amputations were not available in 2004, and thus, we were unable to examine whether the amputation level had improved.

Regarding retinopathy, another microvascular complication, the present study also found an improvement with a significant reduction in both the prevalence of proliferative retinopathy and visual acuity below 0.3. A recent systematic review included studies reporting incidence and progression rates of proliferative diabetic retinopathy and sight‐threatening diabetic retinopathy, covering the period 1980 to 2016.^28^ This review reports that the incidence of both proliferative retinopathy and sight‐threatening retinopathy was reduced by twofold to threefold over the last three decades. In the present study, covering a 15‐year period, we found that the prevalence of proliferative retinopathy and visual impairment decreased twofold, thus corroborating the review. The review suggests that these improvements are explained by better blood pressure and glycaemic control, as well as improvement in diabetic eye screening. Furthermore, the introduction of anti‐vascular endothelial growth factor treatment for diabetic eye disease might play a role.^29^

We found no statistically significant reduction in the prevalence of macrovascular complications over the 15‐year period. These findings are contrary to studies of people with diabetes in general, as well as people with diabetic nephropathy without ESRD, reporting improved prognosis including reduced risk of macrovascular complications as a result of multifactorial treatment.^6^, ^7^, ^8^, ^9^, ^30^ However, in the present study, the mean age was increased by approximately 6 years, and the prevalence of myocardial infarction was reduced from 32% to 20%, indicating a trend towards improvement in terms of cardiovascular complications, although neither was statistically significant. The prevalence of stroke remained at 22%, possibly explained, at least partly, by the high prevalence of atrial fibrillation or flutter (at 31%).

To some extent, the lack of reduction in the prevalence of macrovascular complications in people receiving dialysis might be explained by our limited understanding of the impact of the various risk factors and the optimal treatment in the dialysis population, hindering an effective and comprehensive strategy for preventing complications in this population. This suggests that prevention of complications should be emphasized before people reach ESRD and highlights the importance of aggressive early risk factor modification.

With regard to foot ulcers, our findings suggest that previous foot ulcer should be considered the most important risk factor for new ulceration. Furthermore, our data provide evidence of a positive effect from introducing routine foot check‐ups in our dialysis centre with a significant decrease in current foot ulcers over a 15‐year period. Thus, routinely foot check‐ups in the dialysis unit with increased emphasis on individuals with previous foot ulcer regardless of diabetes status is recommended in order to prevent new ulceration.

There are some limitations to this study that are inherent in cross‐sectional audits of medical records. The cross‐sectional design limits inference from causal relationships, and the data are limited to what is documented in the individuals’ medical records. Our results regarding changes in complications should be interpreted cautiously due to differences in included individuals with diabetes. In 2004, the examination included a questionnaire by which only individuals answering the questionnaire were included, whereas in 2019, all individuals with a diagnosis of diabetes were included in the analysis. However, clinical characteristics and the complication rate were similar between respondents and non‐respondents in 2004, and the inclusion criteria were otherwise equivalent. Another limitation concerns the difference in proportion of type 1 and type 2 diabetes between the two cohorts, as the prevalence of micro‐ and macrovascular complications might be different in individuals with type 1 and type 2 diabetes, respectively. Thus, it would have been relevant to perform analyses for type 1 and type 2 diabetes separately, but we only had sufficient data to perform separate analyses regarding angina pectoris and stroke. Finally, an important limitation of this study was the small sample size, and our findings should be interpreted with caution.

In conclusion, individuals with diabetes receiving dialysis had a higher prevalence of foot ulcer, lower‐extremity amputation and myocardial infarction compared to individuals without diabetes. Previous foot ulcer was the most important risk factor for current foot ulcer, while diabetes and male sex were important risk factors for lower‐extremity amputation.

## CONFLICTS OF INTEREST

The authors have no conflicts of interest to declare for this study.

## AUTHOR CONTRIBUTIONS

DHK, TPA, VRS, BFR and MH designed the study. DHK and TPA collected the data, performed data analysis and wrote the first draft. All authors participated in the analyses and interpretation of data, and in the critical review of the manuscript. All authors approved the final version of the manuscript.

## Data Availability

Data were available on request due to privacy/ethical restrictions.
